# Selective nuclear localization of siRNA by metallic versus semiconducting single wall carbon nanotubes in keratinocytes

**DOI:** 10.4155/fso.15.15

**Published:** 2015-11-01

**Authors:** John Torin Huzil, Evi Saliaj, Marina V Ivanova, Marjan Gharagozloo, Maria Jimena Loureiro, Constanze Lamprecht, Andreas Korinek, Ding Wen Chen, Marianna Foldvari

**Affiliations:** 1 School of Pharmacy, University of Waterloo, 200 University Avenue West, Waterloo, Ontario, N2L 3G1, Canada; 2 Canadian Centre for Electron Microscopy, McMaster University, 1280 Main St. W, Hamilton, ON L8S 4L8, Canada

**Keywords:** chirality, drug delivery, keratinocytes, metallic nanotubes, semiconducting nanotubes, single wall carbon nanotubes, siRNA transfection

## Abstract

**Background:**

The potential use of carbon nanotubes (CNTs) in gene therapy as delivery systems for nucleic acids has been recently recognized. Here, we describe that metallic versus semiconducting single-wall CNTs can produce significant differences in transfection rate and cellular distribution of siRNA in murine PAM212 keratinocytes.

**Results/Methodology:**

The results of cell interaction studies, coupled with supportive computational simulations and ultrastructural studies revealed that the use of metallic single wall CNTs resulted in siRNA delivery into both the cytoplasm and nucleus of keratinocytes, whereas semiconducting CNTs resulted in delivery only to the cytoplasm.

**Conclusion:**

Using enriched fractions of metallic or semiconducting CNTs for siRNA complex preparation may provide specific subcellular targeting advantages.

## Abstract (lay abstract)

Carbon nanotubes are a novel class of nanobuilding blocks extensively investigated as delivery systems for therapeutic RNAi to silence gene expression in the cell cytoplasm and nucleus. It was previously shown that the delivery properties of single wall carbon nanotubes (SWNTs) are influenced by their varied physicochemical characteristics, however, the effect of chirality and electronic properties of SWNTs have not been studied with respect to their role in cellular transfection. To the best of our knowledge, this is the first study that shows using metallic or semiconducting SWNTs for siRNA complex preparation may provide specific subcellular targeting advantages.

**Figure 1.**
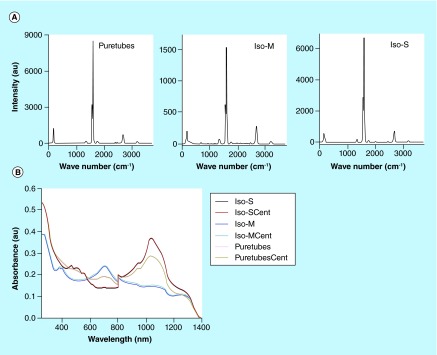
**Raman spectra of the three single wall carbon nanotubes [47], and UV-Vis-NIR spectra of the three single wall carbon nanotubes dispersed in 0.1% (w/v) gemini surfactant solution before and after centrifugation.** Cent: Centrifuged sample; M: Metallic; S: Semiconducting.

**Figure 2.**
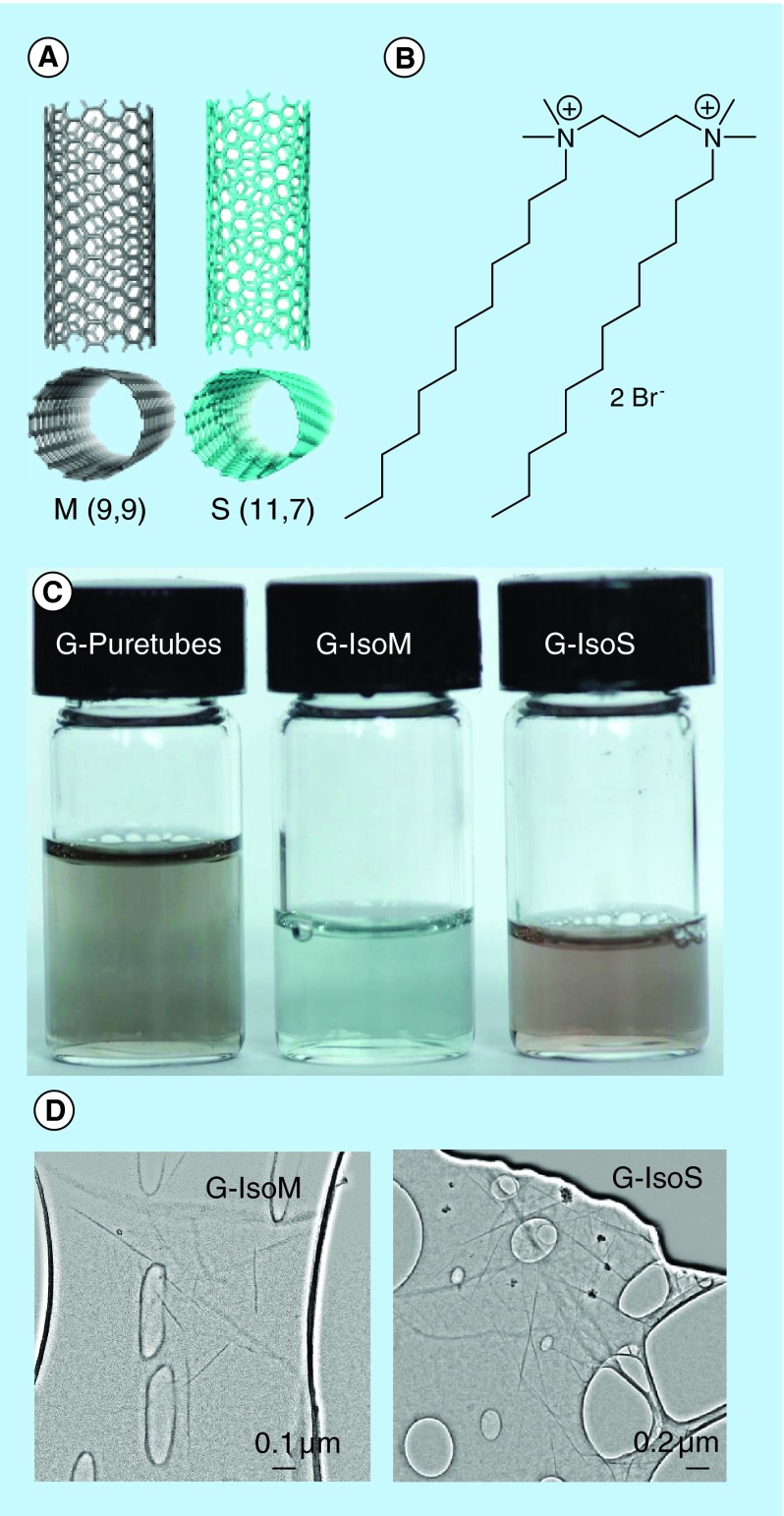
**Characteristics of gemini 12–3–12 surfactant modified single wall carbon nanotubes-siRNA complexes.** **(A)** Computer-generated images of representative M (9,9), and S (11,7) single wall carbon nanotubes; **(B)** structure of gemini surfactant 12–3–12; **(C)** puretubes, Isonanotubes-M and Isonanotubes-S single wall carbon nanotubes (0.1 mg/ml) dispersions in 0.1% (w/v) 12–3–12 G; **(D)** high-resolution electron micrographs of gemini-Isonanotubes-M and gemini-Isonanotubes-S complexes showing individually dispersed nanotubes. G: Gemini surfactant; M:Metallic; S: Semiconducting.

**Figure 3.**
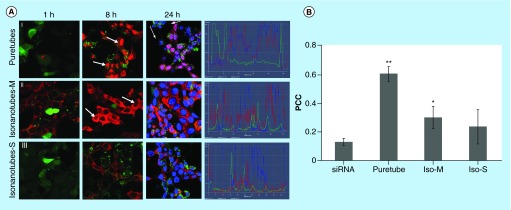
**Uptake of G-single wall carbon nanotubes-siRNA complexes into PAM212 keratinocytes.** **(A)** Cells were imaged using confocal microscopy following 1, 8 and 24 h of incubation (columns). (I) Puretubes; (II) IsoM; and (III) IsoS complexes. Column 4 shows the corresponding profile view of fluorescence distribution along the indicated red line section of the 24 h micrographs. Fluorescence in columns one and two is as follows; red – siGLO-RNA, green – calcein viability stain. In column three, blue fluorescence is pseudocolor – DRAQ5 nuclear/DNA stain, pink – colocalization (red and blue). Arrows indicate accumulation in the nuclei/nucleoli. Bar: 20 µm for all micrographs. **(B)** Colocalization analyses in confocal images of cells using the JACoP plugin of ImageJ. The PCC was calculated for at least five images per treatment and the average calculated values for each condition were plotted on the histogram (error bars represent SD). **Significant in comparison with siRNA (image shown in Figure 4B: free siRNA, 24 h), **p value < 0.001, *p < 0.05. PCC: Pearson's correlation coefficient.

**Figure 4.**
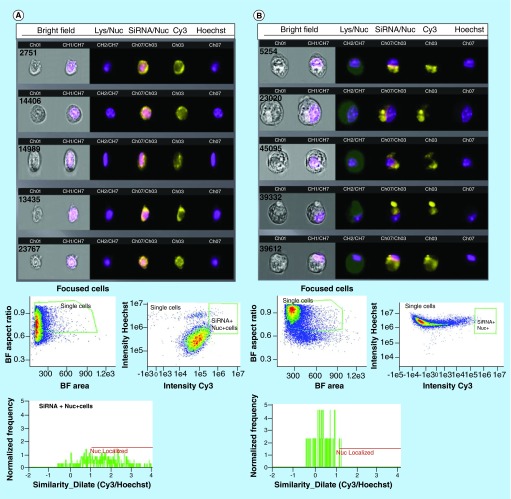
**Imaging flow-cytometry analysis of G-single wall carbon nanotubes-siRNA distribution in PAM212 keratinocytes.** ImageStream multispectral imaging of PAM212 keratinocytes incubated with G-IsoM-siRNA **(A)** and G-IsoS-siRNA **(B)** complexes indicated colocalization of IsoM-siRNA with the nucleus. Similarity algorithm of the IDEAS software was used to measure the spatial colocalization of siRNA with the nuclei of cells where colocalization is present when a score between 1 and 2, or higher is obtained. The Mean Similarity Score for IsoM and IsoS complexes was 1.487 and 0.2983, respectively, after incubation of cells with the complexes at 37°C. BF: Bright field; IsoM: Isonanotubes-metallic; IsoS: Isonanotubes-semiconducting.

**Figure 5.**
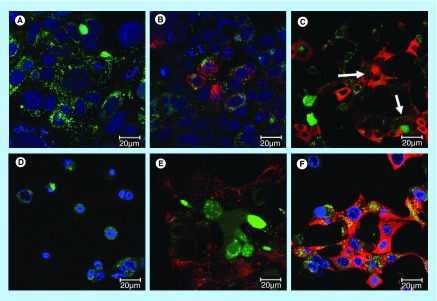
**Uptake of siRNA into PAM212 keratinocytes in control preparations.** Conditions for transfection are identical to those described in Figure 2. **(A)** no treatment, 24 h; **(B)** free siRNA, 24 h; **(C)** G-IsoM/IsoS 30:70-siRNA, 24 h (arrows indicate uptake into the nuclei); **(D)** free 12–3–12 gemini surfactant, 24 h; **(E)** siRNA in 0.1% (w/v) 12–3–12 gemini surfactant solution, 1 h; **(F)** siRNA in 0.1% (w/v) 12–3–12 gemini surfactant solution, 24 h.

**Figure 6.**
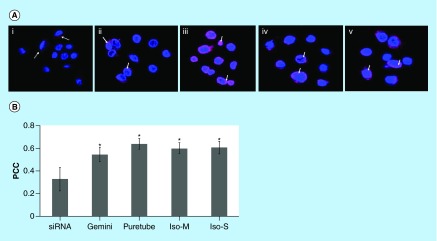
**Uptake of G-single wall carbon nanotubes-siRNA complexes into purified nuclei.** **(A)** Nuclei were imaged using confocal microscopy following 24 h of incubation with (i) free siRNA, (ii) gemini-siRNA, (iii) G-Puretubes-siRNA, (iv) G-Isonanotubes-M-siRNA and (v) G-Isonanotubes-S-siRNA complexes. Dashed arrows represent siRNA (red) outside of nucleus and bold arrows represent colocalization of siRNA into the nucleus (pink). **(B)** Colocalization analyses in confocal z-stack images of nuclei using Huygens Pro software. PCC was calculated for at least five images per treatment and the average calculated values for each condition were plotted on the histogram (error bars represent ±SD). *Significant in comparison with free-siRNA, p value < 0.05. Bar: 10 μm for all micrographs. Gemini: Gemini surfactant; IsoM: Isonanotubes metallic; IsoS: Isonanotubes semiconducting; PCC: Pearson's correlation coefficient.

**Figure 7.**
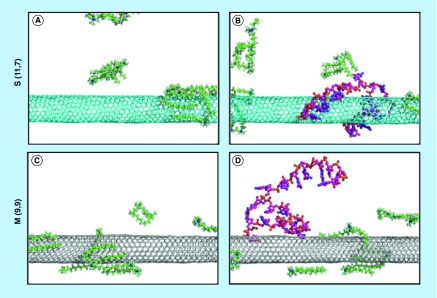
**Simulation of G-single wall carbon nanotubes-siRNA complexes.** Packing of gemini surfactant 12–3–12 on the surface of **(A)** S (11,7) and **(C)** M (9,9) single wall carbon nanotubes. Binding of siGLO-RNA onto the surface of **(B)** the gemini functionalized S (11,7) and **(D)** M (9,9) single wall carbon nanotubes following 10 ns of molecular dynamics. M: Metallic; S: Semiconducting.

**Figure 8.**
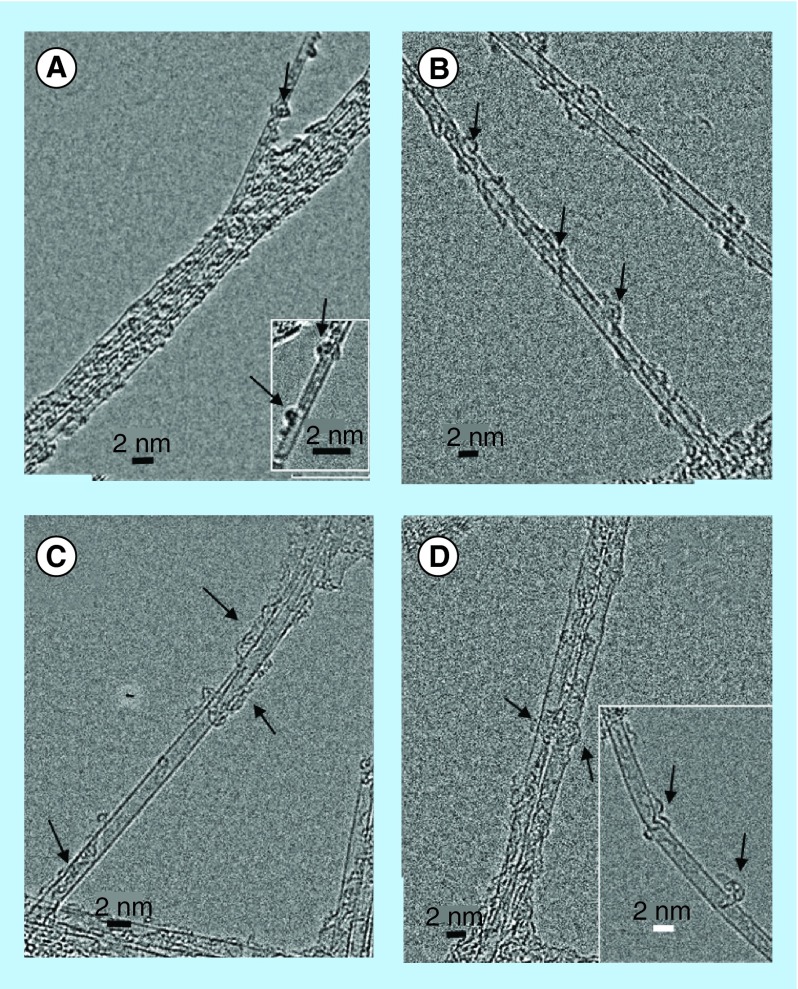
**High-resolution transmission electron micrographs of single wall carbon nanotubes-siRNA complexes.** **(A)** Semiconducting G-Isonanotubes-S complex and **(C)** metallic G-Isonanotubes-M complex and **(B)** semiconducting gemini-IsoS-siRNA and **(D)** metallic G-Isonanotubes-M-siRNA. Insets in **(A)** and **(D)**, represent additional images taken for those samples. Arrows indicate the wrapping pattern of gemini surfactant or siRNA around **(A&B)** semiconducting nanotubes and **(C&D)** metallic nanotubes.

**Figure 9.**
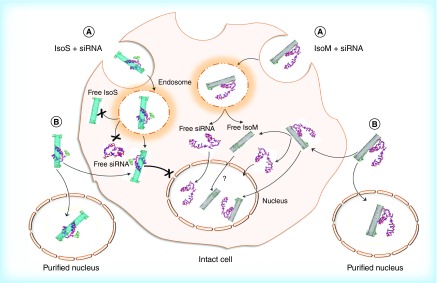
**Hypothesized cellular uptake of metallic Isonanotubes-M-siRNA and semiconducting Isonanotubes-S-siRNA complexes, the release of siRNA inside the cell and its nuclear translocation based on consideration of experimental data.** **(A)** Endocytosis-dependent and **(B)** endocytosis-independent pathways have been postulated as possible uptake mechanisms. After internalization via pathway A or B, the endosomal or cytosolic environment favors the dissociation of siRNA from IsoM, but not IsoS, due to the weak noncovalent interaction between siRNA and IsoM single wall carbon nanotubes. IsoS nanotubes are wrapped more tightly by siRNA and it may not dissociate from IsoS nanotubes inside the cell after either endosomal escape or nonendocytotic uptake. The IsoM-siRNA complex is taken up by a nonendocytotic process and the released siRNA from IsoM after endosomal escape could translocate to the nucleus. Contrary to the IsoM complex, the delivery of IsoS-siRNA complex into the nucleus is inhibited in intact cells, possibly through an unknown cytoplasmic pathway which is not present in purified nuclei. In the absence of specific labeling of the nanotubes themselves, the nuclear translocation of free IsoM nanotubes is uncertain and will need further examination. IsoM: Isonanotubes metallic; IsoS: Isonanotubes semiconducting.

Gene silencing by siRNA is a powerful approach for the treatment of many diseases, including many types of cancer, neurodegenerative, metabolic disorders and infectious diseases [1,2]. siRNA-based therapeutics are an important pharmaceutical approach providing high specificity at very low concentrations [3–6], but new strategies for safe and effective siRNA delivery are still needed. RNA interference mechanisms include gene silencing at two levels: post-transcriptionally in the cytoplasm, the site where the RNA-induced silencing complexes are located; and transcriptionally in the nucleus (recent discoveries indicate the presence of RNA-induced silencing complexes in the nucleus) [7,8].

Many of the challenges in achieving maximum RNAi are related to delivery issues, such as siRNA transport across the cellular membrane and/or translocation into the cytoplasm or nucleus following endocytotic uptake. Carbon nanotubes (CNTs) are a novel class of nanobuilding blocks extensively investigated as pharmaceutical delivery systems and may be useful as siRNA transfection agents [9–12]. While a detailed mechanism describing CNT interactions with cells *in vitro* is still under investigation, there is mounting evidence that they are taken up into cells not only through endocytosis [13,14], but also by sliding through the cell membrane, like a nanoneedle, passively entering the cytoplasm through an energy independent mechanism [15–18]. This bifunctional entry mechanism may provide significant advantages over other delivery systems, such as lipid vesicles and polymers, that are frequently used for siRNA transfection [19,20].

The majority of studies indicate cytoplasmic localization of CNTs [14,15,21–34]; however, some studies showed both cytoplasmic and nuclear presence [13,35–40]. For example, Kostarelos *et al*. [15] demonstrated the uptake of seven different functionalized CNTs in six different cell lines and their localization to the perinuclear region of the cytoplasm. Whereas in a study by Cheng *et al*. [13], fluorescein isothiocyanate (FITC)-labeled PEGylated SWNTs were shown to accumulate in the nucleus, predominantly the nucleoli, of several mammalian cell lines of various histological origins and transformation states. Bhirde *et al*. [40] and Porter *et al*. [38,39] reported nuclear translocation of COOH-functionalized SWNTs using human mesenchymal stem cells. Although the relationship between SWNT properties and cellular uptake has been extensively investigated, a complete understanding of the intracellular fate of SWNTs is still lacking and more importantly, the effect of SWNT electric properties has not been examined thus far.

The delivery properties of CNTs are influenced by their varied physicochemical characteristics. Studies have demonstrated that the nature (single-, double- or multiwall), length, diameter and purity all play a role in CNT entry into cells [10,41], while the effect of chirality is not as clear. Published studies typically used chiral mixtures of electronically semiconducting and metallic nanotubes [42,43] due to the nature of the processes involved in SWNT synthesis. While it is clear that CNT chirality can dramatically influence interactions with nucleic acids [44,45], for example, the electronic properties of metallic and semiconducting CNTs have been exploited to facilitate their separation using DNA-assisted dispersion [46], the effect of chirality has not been studied with respect to its role in cellular transfection.

The objective of the research described in this manuscript was to determine whether siRNA delivery by SWNTs is associated with specificity with respect to intracellular localization and whether chirality influences cytoplasmic versus nuclear accumulation. Here, we describe the results of cell interaction studies, coupled with supportive computational simulations and ultrastructural studies that demonstrate the distinct cellular distribution behavior of metallic versus semiconducting SWNTs during cellular transfection, and a potential correlation with the binding of siRNA to SWNTs in the complex.

## Methods

### Materials

Puretubes, Isonanotubes-M (IsoM, 98% metallic) and Isonanotubes-S (IsoS, 99% semiconducting) SWNTs of similar lengths were purchased as surfactant-eliminated powders from NanoIntegris Inc. (IL, USA) and further characterized (Table 1). siGLO Lamin A/C control cytoplasmic siRNA labeled with DY-547 (since its absorption spectrum is comparable to Cy3 dye, it is referred to as Cy3 in the text) (λ_ex_ = 557 nm/λ_em_ = 570 nm) was obtained from Dharmacon/Thermo Scientific (MA, USA). Nanotube dispersions in 1,2-dichlorobenzene [47] and in 0.1% (w/v) 12–3–12 gemini surfactant solution (before and after centrifugation at 15,000 x *g* for 15 min) were characterized by UV-Vis-NIR spectral scans between 200–1400 nm (Cary 5000 spectrophotometer, Varian Inc., CA, USA), Raman spectroscopy [47] (Figure 1), thermogravimetric analysis [47] and electron microscopy. Gemini surfactant 12–3–12 (1,3-propanediyl-bis(dimethyldodecylammonium) dibromide) from the dicationic *N,N*-bis(dimethylalkyl)-α,ω-alkanediammonium surfactant family was synthesized as previously described [48,49]. The general structure is abbreviated as the m-s-m series where m and s refer to the number of carbon atoms in the alkyl tails and in the polymethylene spacer group, respectively, and X is the counter ion Br (Figure 2B).

### Raman spectroscopy

CNTs were characterized in their dry state using a Senterra Dispersive Raman Spectrometer (Bruker Optics, ON, Canada), with a 532 nm Nd:YAg laser at room temperature. The laser beam was focused onto the sample with a 20× objective and data were collected from 70 to 3700 cm^-1^ at a power of 20 mW. All spectral analyses were performed using OPUS 6.0 software (Bruker Optics). Spectra were taken from three separate regions of the powder sample and overall average spectra were generated. Chiral species in the SWNT preparations were determined from the radial breathing mode frequency peaks (ω_RBM_) (100–350 cm^-1^) as previously described [50,51].

### Nanotube siRNA complexation

Gemini 12–3–12 surfactant modified SWNTs (G-SWNTs) were prepared by sonication of 0.01 mg/ml SWNT in 0.1% (w/v) surfactant at room temperature using the combination of cuphorn closed vessel sonication (S-4000; Misonix Ultrasonic Liquid Processors, NY, USA) for 1 h and bath sonication for 6 h as previously described [47]. One dose of 100 µl SWNT complexes with siGLO-RNA (G-SWNT-siRNA complexes) were formed by adding 10 µl of 5µM (50 pmole/well) siGLO-RNA solution to 90 µl of G-SWNTs at room temperature.

### ζ potential measurement

ζ potential measurements were performed using laser Doppler micro-electrophoresis with Zetasizer Nano ZS Model ZEN3600 (Malvern Instruments Ltd., Worcestershire, UK). The samples were analyzed in folded capillary DTS1060/DTS1061 cells using the Smoluchowski approximation model with Zetasizer software 7.10.

### Transfection & cellular distribution

Murine PAM212 keratinocytes (kindly provided by Dr. S Yuspa, NCI, MA, USA) were subcultured into 50 mm glass bottom Petri-dishes (MatTek Corp., Ashland, MA, USA) to an initial total of approximately 1 × 10^6^ cells/dish in a final volume of 3 ml MEM/EBSS (HyClone, Thermo Scientific, UT, USA) and heat inactivated FBS (Sigma-Aldrich, Oakville, Canada). Cells were then incubated at 37°C, in 5% CO_2_ overnight to allow attachment to the dish prior to treatment with SWNT complexes. When the cells reached 40–65% confluency, they were treated with the following SWNT complex formulations (100 µl per well): G-Puretubes-siRNA; G-IsoM-siRNA; and G-IsoS-siRNA. Once each SWNT complex was applied, the uptake of siRNA was determined at 1, 8 and 24 h time points, using an LSM 710 confocal microscope (Carl Zeiss, Toronto, Canada) and at each time point counterstained with calcein AM viability dye (Life technologies). DRAQ5 (λ_ex_ = 646nm/λ_em_ = 697nm, Biostatus Ltd., Leicestershire, UK) nuclear stain was used at the 24 h time point only.

### Flow cytometry analysis

For imaging flow cytometry analysis using the ImageStream Mark II imaging flow cytometer (Amnis, EMD Millipore, MA, USA), PAM212 keratinocytes (1 × 10^6^ cells/well) were cultured into 6-well plates in a final volume of 2 ml MEM/EBSS complete media overnight prior to treatment with SWNT complexes. The day after, cells were treated with 100 μl of G-IsoM-siRNA, G-IsoS-siRNA complexes and the nuclear uptake of siRNA was evaluated after 24 h incubation at 37°C and 5% CO_2_. At the end of incubation, cells were trypsinized and centrifuged at 200 × *g* for 5 min and then washed once with phosphate buffer saline (PBS). Cells were then resuspended in 1 ml of PBS and stained with 5 µg/ml Hoechst 33342 (λ_ex_ = 350 nm/λ_em_ = 461 nm, Life Technologies, Burlington, ON, Canada) nuclear stain for 15 min. The cells were then washed with PBS and fixed in 100 µl formaldehyde fixation medium (Life Technologies). In total, 10,000 events were collected for all samples using the imaging flow cytometer; Cy3 was excited at 405 nm in channel 3 (560–595 nm) Hoechst 33342 in channel 7 (430–505 nm) and brightfield was emitted in channel 1. Single color controls were run for Cy3 and Hoechst 33342 to correct for spectral cross-talk within the multispectral experimental data. A compensation matrix was derived using the single color controls that were acquired under the identical experimental conditions. To detect siRNA nuclear colocalization, cell populations were hierarchically gated for single cells that were in focus and were positive for both Cy3 and Hoechst 33342 DNA staining. Post-acquisition spectral compensation and data analysis was performed using the IDEAS^®^ image analysis software package (Amnis Corp.). Images were analyzed for the degree of colocalization using the IDEAS™ similarity bright detail (SBD) feature. SBD of the Cy3/Hoechst 33342 images was calculated and images with median nuclear localization score higher than 1 were considered positive for nuclear localization.

### Nuclei isolation & siRNA treatment

Nuclei of PAM212 keratinocytes were isolated using the nuclear isolation kit Nuclei EZ Prep (Sigma-Aldrich) according to the manufacturer's instructions. The purity and integrity of isolated nuclei were confirmed by light microscopy following trypan blue staining. The isolation procedure yielded intact nuclei of at least 85% purity. Isolated nuclei (1 × 10^5^/ml) were resuspended in MEM/EBSS basic media and treated with siRNA-SWNT complexes (100 μl dose/well) in glass bottom 24-well plates (MatTek Corp.). After 16 h incubation at 37°C, the translocation of siRNA to purified nuclei was evaluated by confocal microscopy. Stacks of images consisting of 25–40 slices were collected from nuclei and deconvolved with Huygens Pro software (SVI Huygens Pro 4.3.1 P3, The Netherlands). To estimate the colocalization between siRNA and nuclei, a minimum of five image stacks were evaluated using Huygens Pro software for Pearson's correlation coefficient (PCC) for each channel.

### Electron microscopy

SWNT dispersions were pipetted onto 300 mesh holey carbon coated copper grids (SPI Supplies, PA, USA) and left on for 30 s. The excess sample was drained off with filter paper and the grid dried. To remove excess surfactant matrix surrounding the nanotubes, the grids were washed by applying seven consecutive ultrapure water droplets onto the grid, draining off with filter paper. The nanostructures in the samples were examined with a FEI Titan 80–300 high-resolution (point-to-point resolution 2Å) transmission electron microscope (HRTEM) (FEI Company, Eindhoven, the Netherlands) equipped with a CEOS hexapole image Cs corrector and a Gatan Ultrascan CCD camera at the Canadian Centre for Electron Microscopy at McMaster University (Ontario, Canada). The accelerating voltage was 80 kV. The illumination background on the images was corrected by calculating a local background of the micrograph using a 20 × 20 pixel real space kernel. This background was subtracted from the original micrograph.

### Molecular modeling

The secondary and tertiary structure of siRNA was modeled using the software package Assemble [52]. Secondary structure interactions were determined using RNAplot [53], followed by tertiary structure prediction through homology modeling. Two SWNT models were then generated using Visual Molecular Dynamics (VMD) nanotube modeler [54]. These included: a metallic nanotube (n = 9, m = 9) and a semiconducting nanotube (n = 11, m = 7), both having a diameter of 1.2 nm and length of 50 nm. Each SWNT was placed in a periodic simulation box with dimensions 50 × 50 × 50 nm and solvated for an initial minimization step. Production molecular dynamics runs of a SWNT with 15 gemini surfactant molecules and a single siRNA molecule were then performed using GROMACS 4.5.5 [55] and a modified version of the OPLSaa force field accounting for the SWNT carbon and nucleic acid atom parameters. A steepest descents minimization, followed by 10 ns of constant NPT molecular dynamics was then performed.

### Statistical analysis

Statistical analysis of the colocalization data was carried out using GraphPad Prism software (Intuitive Software for Science, CA, USA). One way ANOVA test followed by Dunnet post hoc test was used for the analysis between different group experiments. A p-value of less than 0.05 was considered statistically significant.

## Results

### Physicochemical properties of SWNT-siRNA complexes

To develop siRNA-SWNT complexes we used commercially available nanotubes without further purification. Puretubes contain a mixture of chiral species, while the other two (IsoM and IsoS) SWNTs contain purified fractions of metallic or semiconducting nanotubes, respectively (Figures 1 & 2; Table 1). Physicochemical parameters are shown in Table 1, including chirality for the main resonant nanotubes, which was determined from the respective Raman spectra (Figure 1). The UV-Vis-NIR spectral scans of the four SWNT dispersions in 1,2-dichlorobenzene [47] and in 0.1% w/v 12–3–12 gemini surfactant were obtained to characterize the nanotubes used in the study. IsoM and IsoS nanotubes showed the characteristic metallic absorbance peak centered at 700 nm and semiconducting peak at 1050 nm, respectively (Figure 1). The Puretubes SWNTs, being a mixture of multiple chiral species, exhibited both peaks with a metallic/semiconducting ratio of approximately 30/70, a typical chiral distribution normally observed in bulk SWNT samples. Gemini surfactant-dispersed SWNTs (G-SWNTs) at 0.1 mg/ml concentration were visibly clear solutions (Figure 2C). Electron microscopic observations (Figure 2D), indicated the presence of individually dispersed nanotubes and some small bundles. G-SWNTs with siRNA (G-SWNT-siRNA complexes) were prepared by adding siRNA to the respective SWNT dispersion. Complexation of G-Puretubes, G-IsoM, G-IsoS SWNTs with siRNA produced uniform dispersions, with an average ζ-potential of +50.8 ±5.2; +55.8 ±2.3; +54.3 ±2.2; +59.0 ±5.8 mV (n = 3), respectively.

### Subcellular distribution of SWNT-siRNA complexes

Following the incubation of G-SWNT-siRNA complexes with PAM212 keratinocytes, Puretubes complexes demonstrated significant differences in both the rate of transfection and the overall pattern of cytoplasmic and nuclear siRNA distribution (Figure 3). Binding of G-SWNT-complexes to the cellular membrane was detectable after 1 h of incubation, especially for Puretubes and IsoM, and to a lesser extent by IsoS SWNTs (Figure 3A, Column 1). The binding of siRNA to cell membranes and subsequent uptake into the cytoplasm was significantly greater only for the IsoM SWNT complexes, 1 h post dosing (Figure 3A[II]). Within 8 h following dosing, robust transfection of the cells was observed for all three of the CNT complexes (Figure 3A, Column 2). Red fluorescence signals were reproducibly detected in the cytoplasm of keratinocytes, while accumulation in the nuclei was only observed for the Puretubes, and IsoM complexes (Figure 3A[I&II], Column 2). Furthermore, Puretubes complexes showed accumulation of siRNA in the nuclei, the IsoM complexes seemed to be more selective toward nucleoli (Figure 3A[I&II] Column 2, arrows). When cells were counter-stained with DRAQ5 at the 24 h time point, a similar, CNT dependent, cytoplasmic and nuclear distribution pattern of siRNA to the 8 h time point was observed (Figure 3A, Column 3). There was no signal in the nuclei of cells treated with the G-IsoS complexes (Figure 3A[III], Column 3). The corresponding intensity curves for the three treatments at 24 h (for the indicated profile line within the images) confirm the nuclear localization of siRNA (red) and nuclear staining (blue) for G-Puretubes and G-IsoM complexes, and show the absence of siRNA in the nuclei of cells treated with the G-IsoS complex (Figure 3A, Column 4). These observations suggested that the electronic properties of IsoM SWNTs played a significant role in siRNA delivery into the nucleus. After 24 h, there was evidence of a decrease in red fluorescence intensity from the nuclei of cells treated with the Puretubes complex, as compared with the 8 h time point (Figure 3A[I], Columns 2 and 3). To confirm that the nuclear translocation of siRNA was SWNT-mediated, localization of siRNA to the nucleus was calculated in confocal images taken after 24 h incubation (Figure 3B). Correlation analysis between the fluorescence intensities observed for nuclei (DRAQ5, blue) and siRNA (Cy3, red) were expressed as Pearson Correlation Coefficients (PCC). A strong localization of siRNA within nuclei was observed for the G-Puretubes when compared with free siRNA and G-IsoM complexes (p < 0.001 and p < 0.05). Cells treated with G-IsoM-siRNA complexes also showed significantly higher accumulation of siRNA inside the nuclei compared with free siRNA. However, no significant siRNA colocalization in the nucleus was noted in cells treated with G-IsoS-siRNA complexes for 24 h. Additional studies using imaging flow cytometry with quantitative image-based colocalization of siRNA and nucleus were performed (Figure 4). Analysis of PAM212 keratinocytes incubated with G-IsoM-siRNA and G-IsoS-siRNA complexes at 37°C indicated colocalization of IsoM-siRNA with the nucleus with the mean similarity score for nuclear co-localization of 1.487 and no colocalization with G-IsoS-siRNA complexes (score 0.2983).

### Assessment of controls

No treatment samples showed green fluorescence due to the calcein viability stain (Figure 5A) and no red (Cy3) fluorescence. Free siRNA was taken up very slowly (red cytoplasmic fluorescence was only noticeable 24 h after dosing) and to a limited extent into keratinocytes and there was no signal present in the nuclei (Figure 5B). G-IsoM and G-IsoS complexes were combined at a 30:70 ratio (approximately the same ratio found in the Puretubes sample [47]) and subsequently complexed with siRNA. Incubation with G-IsoM/Iso-S 30:70 complex resulted in the delivery of siRNA into both the cytoplasm and nucleoli of cells, producing a similar pattern to that observed for the G-IsoM complexes alone (Figure 5C). Gemini surfactant alone at 0.1% (w/v) concentration (same as the starting concentration used to prepare G-SWNT complexes) did not show any red fluorescence, however, there were signs of cellular toxicity (rounding of cells at 24 h) (Figure 5D). When siRNA was introduced with 0.1% (w/v) gemini surfactant, we observed an overall increase in cytoplasmic uptake, which may be due to the capacity of gemini surfactant molecules to influence overall membrane permeability at both 1 h and 24 h incubation (Figure 5E&F). However, unlike cells exposed to the G-Puretubes, G-IsoM or the IsoM/IsoS 30:70 complexes, there was no evidence of siRNA translocation into the nucleus and the level of cellular toxicity was lower at both time points compared with gemini surfactant alone (Figure 5D). Since gemini surfactant is present in all formulations in both SWNT-bound and unbound form, during cell interaction the unbound form may influence the integrity of the cell membrane to some degree. However, free gemini surfactant was likely present in only a small fraction compared with the bound form, since there was no noticeable influence on early siRNA delivery by G-SWNT complexes (Figure 3A), as compared with gemini surfactant with siRNA where the gemini surfactant was present at 0.1% (w/v) concentration (Figure 5E&F).

In order to elucidate whether cytoplasmic factors were involved in the delivery of G-SWNT-siRNA complexes to the nucleus, the degree of nuclear siRNA colocalization was also assessed in purified nuclei after 24 h incubation with SWNT complexes (Figure 6). G-siRNA complexes as well as G-SWNT-siRNA complexes showed significant (p < 0.05) accumulation in the nuclei compared with the free siRNA.

### Modeling & high-resolution TEM imaging of SWNT interactions with siRNA

The experimentally observed differences in cellular siRNA distribution led us to use complementary techniques to better understand siRNA interactions induced by the electronic structure of SWNTs and the possible role it may play on intracellular fate. Initial computer simulations examining bare SWNTs and their interactions with siRNA were in agreement with previous computational and experimental results that demonstrated CNT-siRNA interactions were induced through pi-stacking, resulting in decreased binding to metallic CNTs as a result of lower surface charge [56,57]. We observed that the interaction of gemini surfactants with the SWNT surface exhibited differences between the S (11,7) and M (9,9) species (Figure 2A), which were selected as representative models (Figure 7, Panels A and C, respectively). In these simulations, there were fewer, more disordered interactions with the semiconducting SWNT, while interactions with the metallic SWNT exhibited a lateral, distributed packing over the entire CNT surface. In simulations examining SWNTs, gemini surfactants and siRNA, we observed a decrease in the binding affinity between the metallic SWNT when compared with the semiconducting SWNT. This is best illustrated by the transient interaction between the siRNA and the metallic nanotube, while the siRNA molecule remained bound to the semiconducting nanotube for the duration of the 10 ns simulation (Figure 7B&D, respectively).

High-resolution electron microscopic observations of G-SWNT and G-SWNT-siRNA complexes further confirmed the data from the modeling experiments. The association of gemini surfactants and siRNA with IsoM and IsoS SWNTs is illustrated in significant morphological detail in Figure 8. Both types of nanotubes dispersed into individual or very small bundles of tubes and became coated with gemini surfactant molecules (Figure 8A&C) or gemini surfactant and siRNA molecules (Figure 8B&D). Gemini surfactants appeared to bind to IsoS SWNTs in small group-like patches (Figure 8A), whereas binding to IsoM SWNTs was more evenly distributed on the surface (Figure 8B). Furthermore, the wrapping of the siRNA molecules around the G-IsoS was more continuous (Figure 8C), whereas it showed an intermittent or partial wrapping pattern to G-IsoM (Figure 8D).

## Discussion

Previous reports of siRNA delivery into cells using CNTs have typically employed chemically modified SWNTs [13,15,26,31,40]. Here, we describe the delivery of siRNA into keratinocytes by non-covalently functionalized SWNTs, which allows the preservation of patterned binding, a phenomenon frequently studied between CNTs and nucleic acids. Our results suggest that the siRNA distribution mediated by G-IsoM complexes in the cytoplasm is indicative of a cytoplasmic pattern, followed by nuclear translocation of the siRNA cargo. For G-IsoS complexes, labeled siRNA appeared to remain within the cytoplasm and was subsequently slowly degraded/processed by the cells, as indicated by the decreasing intensity of the red fluorescence over time (Figure 3A[III], Columns 2 and 3). While siRNA did not appear to become translocated to the nucleus by G-IsoS complexes in intact cells, its nuclear colocalization was detected in purified nuclei, suggesting that in whole cells G-IsoS-siRNA complexes were probably prevented from nuclear translocation. Quantitative colocalization analysis of siRNA in purified nuclei also revealed that all G-SWNT-siRNA complexes demonstrated significant translocation to the nucleus as compared with free siRNA. Considering the small diameter of SWNT complexes (Figure 1 & Table 1), one possible mechanism may be passive diffusion through the nuclear pore complex which is estimated to have a diameter of approximately 70 nm both at the cytoplasmic and nuclear openings, and 45–50 nm at its midplane [58].

Based on experimental and computational modeling results, it is evident that binding of siRNA to metallic or semiconducting SWNTs differs as a result of their underlying electronic properties. Generally, siRNA may interact with SWNTs directly even if coated with gemini surfactant or there is a combination of interactions involving the electrostatic binding with the two cationic headgroups of the gemini surfactant already bound to the nanotube surface through its alkyl chain, similarly to reports on other surfactants and phospholipids [59,60] and by direct hydrophobic and van der Waals interactions with the nanotube surface. The difference in binding interactions between siRNA and IsoM or IsoS SWNTs is evident even within this short 10 ns simulation indicating more extensive wrapping of siRNA to semiconducting SWNTs, which ultimately could lead to less effective delivery. A possible model for the fate of metallic and semiconducting SWNT-siRNA complexes is shown in Figure 9. Both metallic and semiconducting SWNTs could enter the cells by either endocytotic or nonendocytotic mechanisms. Once inside the cytoplasm, the lower binding affinity toward metallic SWNTs may assist in the release of the siRNA and its translocation into the nucleus possibly in the presence of free gemini surfactant molecules disassociating from the SWNTs or alternatively the whole G-IsoM-siRNA complex may enter the nucleus. The more extensive association of siRNA with the G-IsoS complexes may not permit the same degree of release of siRNA compared with the G-IsoM complexes and therefore, nuclear uptake is not observed. The direct entry of G-IsoS-siRNA complexes into the nucleus was also not observed, however, the reason for this is not yet clear. Although Puretubes showed a high level of siRNA delivery to the cell cytoplasm, delivery to the nucleus was also more pronounced with Puretubes. Recently published data showed that SWNTs with lower chirality (m,0) penetrate deeper into the cell membrane [61].

## Conclusion

Using enriched fractions of metallic or semiconducting SWNTs for siRNA complex preparation may provide specific subcellular targeting advantages. Here, we have demonstrated that metallic SWNT complexes rapidly, within 1 h, deliver siRNA into keratinocytes and are capable of delivering siRNA into the nucleus, whereas semiconducting SWNTs transport siRNA slowly and to a more limited extent and only to the cytoplasm. These observations suggest that the electronic properties of IsoM SWNTs plays a significant role in siRNA delivery into the nucleus. While the underlying molecular mechanisms associated with this translocation process still need to be determined, it is apparent that this selectivity may provide advantages in the future development of nonviral gene delivery systems.

## Future perspective

Significant progresses have been made in the development of efficient siRNA delivery in non-viral systems, such as cationic lipids and polymers. However, a major problem with these approaches is the off-target effects of siRNA that has to be administered for efficient gene silencing. siRNA conjugation to antibodies or aptamers is a common approach for cell-specific targeted delivery. However, the development of delivery systems for siRNA to specific subcellular compartments such as the cytoplasm, nucleus, lysosome or mitochondria is still in its infancy. Based on what we have observed for CNTs, tuning the physicochemical properties of nanomaterials could be considered as an approach for developing intelligent delivery systems capable not only of cell-specific delivery but also targeting specific subcellular compartments, in particular nucleus, which is the critical step in gene therapy.

**Table 1.** **Physical and physicochemical parameters of single wall carbon nanotubes.**

**SWNT**	**Reported diameter (nm)^†^**	**Calculated diameter (nm)^‡^**	**Mean length (µm)^†^**	**ε_ave_ (ml/cm.mg)^§^**	**Possible Chiralities^#^**	**Impurities (wt%)**		**Manufacturing method**
NanoIntegris PureTubes (batch P09–562)	1.2–1.7	1.44	0.6	53.1 ±2.9	(24,2) S	<0.5^¶^; <5^††^	0.23 Ni	Arc discharge
					(16,10) M		0.05 Y	
					(14,9) S (19,2) S		0.06 Fe	
					(13,8) S			
					(14,4) S			
NanoIntegris IsoNanotubes-M (batch M09–910)	1.2–1.7	1.41	0.4	51.7 ±6.6	(23,5)	<1^¶^; <5^††^	0.07 Ni	Arc discharge
					(14,14) (22,4)		0.38 Y	
					(16,10)		0.72 Fe	
					(13,10) (17,5)		5.29 I	
					(18,0)			
					(12,6)			
NanoIntegris IsoNanotubes-S (batch S09–248)	1.2–1.7	1.56	0.6	54.2 ±3.1	(18,11)	<1^¶^; <5^††^	0.07 Ni	Arc discharge
					(14,12) (12,11)		0.38 Y	
					(12,8) (15,4)		0.72 Fe	
					(13,5)		5.29 I	

^†^Data from NanoIntegris Technical Data Sheet histograms.

^‡^Diameter was calculated [47] using C_1_ = 223.5 cm^-1^?nm, C_2_ = 12.5 cm^-1^.

^§^Extinction coefficient (**ε_ave_**). Data taken from [47].

^#^Chirality for the main resonant nanotubes was determined from Raman spectra according to [50,51].

^¶^Metal catalyst.

^††^Carbonaceous.

M: Metallic; S: Semiconducting; SWNT: Single wall carbon nanotubes

**Executive summary**

Carbon nanotubes (CNTs) are a novel class of nanobuilding blocks extensively investigated as pharmaceutical delivery systems and may be useful as siRNA transfection agents.
The objective of this research was to determine whether siRNA delivery by single wall carbon nanotubes (SWNTs) is associated with specificity with respect to intracellular localization and whether chirality influences cytoplasmic versus nuclear accumulation.
The study presents confocal laser scanning microscopic imaging of purified fractions of metallic and semiconducting SWNT – cell interactions, supportive computational simulations and ultrastructural studies that demonstrate the distinct cellular distribution behavior of metallic versus semiconducting SWNTs during cellular transfection and a potential correlation with the binding of siRNA to SWNTs in the complex.
Metallic SWNT (Isonanotubes M) complexes rapidly, within 1 h, deliver siRNA into keratinocytes and are capable of delivering siRNA into the nucleus, whereas semiconducting SWNTs transport siRNA slowly and to a more limited extent and only to the cytoplasm. These observations suggest that the electronic properties of IsoM SWNTs play a significant role in siRNA delivery into the nucleus.
Tuning the physicochemical properties of nanomaterials is an important strategy for developing intelligent delivery systems capable of not only cell-specific delivery but also targeting specific subcellular compartments, in particular nucleus, which is critical in nucleic acid delivery.
